# Health consequences of cricket – view from South Asia

**DOI:** 10.1186/1755-7682-6-30

**Published:** 2013-07-27

**Authors:** Asfandyar Sheikh, Syed Arsalan Ali, Anum Saleem, Sajid Ali, Syed Salman Ahmed

**Affiliations:** 1 Dow Medical College, Dow University of Health Sciences, Baba-e-Urdu Road, Karachi, Pakistan

## Abstract

Although cricket has origins in the British Empire, it is followed as a religion in South Asia, probably due to the influence of the former during their rule. The sport is equally popular among all groups of the society, and is not subject to gender or age constraints. It marks the epitome of reverence and is considered a battle for self-esteem, not only for those playing, but for those watching as well. The intensity of emotional attachment with this sport renders certain public health benefits as well as drawbacks to the general masses.

## Background

WHO defines health as: “a state of complete physical, mental and social well-being and not merely the absence of disease or infirmity” [1]. Technological advancements in recent decades have witnessed a proportional decline in the levels of physical activity, with the shockwaves of inactivity mostly plaguing the lives of people living in developed countries. For majority of people, only a minimum amount of physical effort is involved in their domestic chores and at work. The “daily morning walk” has become an extinct concept in the modern society. The consequential sedentary lifestyle is considered a major risk factor for a large number of metabolic conditions such as cardiovascular disease, cancer, diabetes and obesity [2,3].

Sports have been used as a means of improving physical, social and spiritual health since ancient times. However, benefits from sports can extend beyond the aforementioned domains, and include other positive impacts related to mental wellbeing and personal development [4]. The sporting sector serves as an unmatched tool for increasing rates of physical activity. As such, sporting ‘mega events’ are typically termed as ‘a unique opportunity to improve public health’ [5]. However, the health consequences of sporting events are still debatable, as no study has succeeded in establishing a positive correlation. For example, a previously published systematic review of literature between 1978 and 2008, has found insufficient evidence to support or refute any health or socioeconomic impacts from major multisport events [6].

In this review, we aim to briefly identify the possible health impacts of sporting activities, with special reference to cricket. The demographic area targeted is South Asia, which is a major hub for cricketing events.

### Sports – a public health perspective

Sports encourage healthy lifestyle choices among people belonging to all ages and genders. They exert beneficial effects on bone metabolism, promote cardiovascular and respiratory health and contribute to improvements in motor and cognitive functions [7-9]. Moreover, they have also been shown to have a major impact on the psychiatric health [10]. There is enough evidence to suggest that physical activity can attenuate the symptoms of depression and melancholia, and can also be used to improve the mental status via mood elevation and induction of self-confidence [11,12].

Regular physical activity reduces the risk of premature mortality [13]. Substantial evidence exists for the beneficial effects of regular exercise on cardiovascular, metabolic and neoplastic disorders [14,15]. It has been estimated that a large proportion of all cancer cases may be prevented by a healthy diet, physical activity and no tobacco use [16]. As such, exercise and sports have long been utilized in the treatment and rehabilitation of non-communicable diseases [17-19]. However, their scope is not limited to amelioration of non-communicable diseases only. Sports have also been used as a didactic tool to raise awareness on communicable diseases such as HIV/AIDS, malaria and tuberculosis in developing countries via large scale advertisement campaigns sponsored by major sporting organizations [20-23].

However sports, analogous to the impressive Dr Jekyll, also have a darker side reminiscent of Edward Hyde. Different kinds of sports can lead to multiple health related problems and varying degrees of injuries [24]. Almost 10-19% acute injuries presenting in the emergency departments in Scandinavian countries are sports injuries, with preponderance of knee and ankle injuries [25]. The highest incidence is seen in adolescents playing sports with demanding physical requirements, such as football, basketball, and cricket. However, reports show that women playing these sports have a three to five times greater chance of contracting a serious knee injury than men [26-28].

Apart from injuries, sport events are also associated with increased risk of exposure to infectious agents [29,30]. Fans travel from different parts of the world to watch their favorite superstars in action. This significantly increases the risk of “imported cases,” which may pose an even bigger threat in congested settings, such as those for spectators in such events. The resultant outbreaks may then become a public health problem, and may pose a significant socioeconomic burden even after the event has ended. These health concerns surround not only the spectators, but also the players, especially those participating in contact sports [31].

### Pediatric and adolescent implications

Experts recommend at least one hour of light physical activity every day for school-going children. Childhood and adolescence are no longer referred to as the healthiest part of a person’s lifecycle. This has been made possible by the lethargic lifestyle prevalent individuals belonging to this age group. Youngsters today are not active enough, as computers and other modern day electronics that compete for their attention. Overweight children generally live more sedentary lifestyles and are socially inert. They have an increased risk for developing serious health conditions such as heart disease, high blood pressure, type 2 diabetes, fatty liver disease, sleep apnea and asthma. Engaging in adequate physical activity is considered an essential component for the maintenance of weight loss and provides many other health benefits for children [32]. Other studies, however, have failed to show lower BMI or fat mass in children who display higher sports participation [33-35]. Personality characteristics, such as achievement, motivation, self-confidence, independence, and one's perceived ability to be active (i.e., self-efficacy), are also associated with physical activity levels [36,37]. It has also been suggested that physical activity, particularly sports participation, may affect the development of self-esteem in adolescents [38].

### Video games

Video game companies usually release new editions of their popular sporting games after major events. Children spend a large amount of time playing these games, especially on the computer [39]. These video games have both positive and negative effects on health. The effects, however, vary immensely depending on the types of games played and the frequency of play. Excessive video game playing may cause black rings under the eyes and frozen shoulders, possibly as a result of inappropriate posture or sleep deprivation [40]. Nocturnal video-game playing, especially those involving stimulating or intense storylines, have been linked to later bedtimes, insufficient sleep, and increased daytime tiredness [41,42]. However, video game playing may also directly be associated with vision problems [43]. Video games have been found to improve cognitive alertness and thus lead to aggression in a study conducted using functional magnetic resonance imaging (fMRI) by Mathiak et al. [44]. Other studies have yielded contradictory results elucidating this link [45-47]. In short, frequent video gaming desensitizes players to its cognitively stimulating effects, and this effect is further translated into their daily lives [48].

The term “exergaming” needs special mention when discussing the beneficial effects of video games. It is used to denote a hybrid of video gaming and exercise. When kids play exergames, they burn more calories than if they were sitting and playing video games. Videogame companies have released several versions of consoles especially designed to promote exergaming. Apart from benefits associated with exergaming, videogames are said to improve the player's manual dexterity and computer literacy.

### Health implications – viewers

As mentioned before, different forms of sporting activities are widely used as an educational tool for arousing awareness against factors influencing communicable diseases such as HIV/AIDS, malaria and tuberculosis via mass media advertisement campaigns [49]. For example, sports events, especially organized by UNICEF, have been used to promote healthy immunization practices against measles in Zambia [50]. However, excessive broadcasting of major sports events has led to the overgrowth of “couch potatoes”. Spending countless hours in front of the television has long been linked with the development of obesity, an important risk factor for diabetes. Although a large part of this is attributable to the sedentary nature of this activity, watching too much television has been found to be an independent risk factor for obesity and other metabolic disorders [51]. Furthermore, other factors such as increase in advertisements for junk food have performed an insidious role, but nevertheless, are equal partners in crime.

Excessive television viewing has other implications beyond disturbing the metabolic status. For example, it has been found to be associated with an increased risk of myopia and other visual abnormalities. Similarly, long periods of television viewing may lead to impingement of neuronal mechanism leading to short attention span and a possibility of developing autism [52]. Furthermore, contemporary facts put forward suggest that television spectacle may have association with the development of Alzheimer's disease [53]. Television viewing has also been implicated in suppressing production of melatonin, a key hormone that has important roles in the immune system, sleep/wake cycle and the onset of puberty [54].

Cricket dominates the spectrum of outdoor games played in South Asia. Therefore, it is only fitting to discuss its health impacts separately.

### Cricket

Although cricket has origins in the British Empire, it is followed as a religion in South Asia, probably due to the influence of the former during their rule [55]. The sport is equally popular among all groups of the society, and is not subject to gender or age constraints. It marks the epitome of reverence and is considered a battle for self-esteem, not only for those playing, but for those watching as well. The intensity of emotional attachment with this sport renders certain public health benefits as well as drawbacks to the general masses.

Cricket matches come in three flavors: test, one-day and twenty-twenty. The former is usually considered a trial of a player’s psychological strength, whereas the latter two usually judge his corporeal strength. During the span of one match, the bowlers, batsmen, fielders and the wicket-keepers are subjected to tremendous physical and mental stress. For example, contrary to its unidentical twin baseball, the bowler in this sport usually bowls from a variable runup. This runup may range from a few feet to several meters. Once the bowler reaches the delivery mark, he must ensure that his arm does not exceed a certain angle, while reaching optimum speeds. He must also maintain certain wrist positions in order move the ball in the air or off the pitch. The complete process, therefore, requires a complex amalgamation of might and dexterity. Similarly, the batsman has to judge the pitch of the ball, and then use his muscles to artistically divert it to his location of choice. He then has to crossover to the other side and complete a run. The fielders have to remain vigilant throughout the course of the innings, and once the ball finds their territory, have to run on to gather it and then throw it back. These, along with the boons similar to those associated with other strategic team sports, imply that each cricket game is an intense and involving experience, and every minute spent on the field requires an intricate balance between the mind and the body.

The Worldcup, which is organized every 4 years, is the major tournament for cricket. This tournament attracts a lot of fans from different countries, and thus provides opportunities for diplomatic and economic benefits. Other formats of cricket are more popular among localized populations. For example, “gully cricket” is a phenomenon not too peculiar to the streets of South Asia. Almost every subcontinental street has witnessed at least one game of this genre. In this format, streets that do not receive a lot of ongoing traffic are used as cricket fields. This format, therefore, serves as a healthy platform for active participation of all individuals in a society, and not just professional cricketers.

### Health benefits of cricket

Cricket has been shown, in various studies, to improve stamina and endurance. Most professional cricketers undergo rigorous training periods before they are considered fit to play [56]. The training exercises that form part of the normal conditioning of these individuals help them attain endurance levels comparable to those of players from other, more intense sports [57]. A complete cricket player plays in different roles throughout his career. Each of these roles requires psychological strength, self-confidence and mental toughness. On one hand, he is a versatile technocrat, with immaculate hand-eye coordination, optimal balance and superior perceptual skills [58]. On the other, he is a strategist and an economist, devising tactics to contain the opponents, while simultaneously guiding his own team mates to achieve the best. On yet another, he is an adamant adversary, showing aggression where it is required, without losing his head in the process.

In 1955, Fletcher conducted an evaluation of the average energy expenditure in test cricket during the 1953 Ashes Test series between England and Australia [59]. He calculated that the mean daily physical activity for an ideal player was 86.4 kcal.m^-2^.h^-1^, corresponding to an energy expenditure of 680 kJ.h^-1^ for an average cricketer having a body surface area of 1.8 m^2^. He concluded that the mean energy expenditure for an average Test cricketer was only slightly more than that achieved while standing, with the expenditure surpassing tennis only while practicing in the nets. This study gives an impression that cricket is a relatively undemanding sport. However, Noakes rebuked this impression, stating that one-day cricket was a far consuming format, which required the players to be athletic [57]. Further analysis of the South African cricket team in the same paper revealed that most of them were proficient in other forms of sports, especially rugby, with many achieving the same level of fitness as professional rugby players.

### Health concerns of cricket

However cricket, once called the “gentleman’s game,” has also been a target of criticism from health circles. For example, professional cricketers spend countless hours in open daylight. Excessive exposure to heat in these individuals increases the risk of dehydration, heat stroke and heat exhaustion, whereas excessive sunlight exposure has been associated with an increased risk of melanoma and other skin cancers [60]. To counter this, most cricketers use different brands of sunscreens, which themselves have health implications [61]. In addition, cricketers have also been known to suffer from depressive and other psychological disorders, with many requiring rehabilitation [62]. Apart from the direct effects on cricketers, inappropriate advertisements in commercial cricket have been criticized in the past. For example, concerns have been raised in the past over the sponsorship of events by a fast-food chain, which may promote unhealthy ingestion of fried foods and thus lead to obesity [63]. Similar concerns have been voiced for alcohol promotion via cricket advertisement [64].

### Cricketing injuries

Injuries sustained during cricketing activities form a major chunk of the health hazards caused as a result of playing the sport. A cricketing injury is defined as ‘any injury or other medical condition that either prevents a player from being fully available for selection for a major match, or during a major match, causes a player to be unable to bat, bowl or keep wicket when required’ [65]. Current reports estimate the incidence of cricket injuries at 2.6/10,000 athlete hours played, with 28.4% to 71.6% of cricketers sustaining between 1.61 and 1.91 injuries per season [66-68]. Lower limb injuries account for 22.8% to 50.0% of the injuries sustained, whereas upper limb injuries are responsible for 19.8 to 34.1% [67-69]. Back and trunk injuries account for about 18% and 33.3% of total injuries, whereas head and neck injuries account for 5.4% to 25% [67-69].

Cricketing injuries range from minor contusions and lacerations to career-threatening deformations. For example, Mark Boucher, the former South African wicketkeeper was forced to retire early from international cricket following a serious eye injury. Graver consequences have occurred to other players, with many not being able to cope with the imposing physiological demands of the game, and some even dying during the course of a match. Fast bowlers and batsmen are especially the endangered species [70]. The pacers are mostly at risk for hamstring injuries, rotator cuff injuries, lower back pain and sprained ankles, whereas batsmen and fielders are mostly threatened by impact injuries caused by the cricket ball [71]. However, the scope of cricket-related injuries is not limited to the players only. Umpires, and even spectators, have suffered fatal injuries as a result of the deadly impact of the murderous cricket ball [61,62].

#### Injuries to bowlers

Bowlers, especially fast bowlers, are at the highest risk of cricketing injuries, with 33.0% to 65.7% sustaining back injuries [68]. Overuse injuries due to repetitive movements are the most common. Bony abnormalities such as spondylosis are often encountered in first-class cricket, which involves long spells of bowling, whereas stress fractures mainly involve the metatarsals, fibula and tibia [72]. The main reasons include improper bowling technique, long spells with repetitive movements and excessive training [73]. Spin bowlers, on the other hand, are subjected to splitting or wearing injuries caused by the seam of the ball [66].

#### Injuries to batsmen, fielders and wicketkeepers

Batsmen and fielders are at an increased risk of impact injuries caused by the incoming ball. These may include fractures, soft tissue injuries as well as orbital injuries [74]. Splenic ruptures have also been reported due to impact of the cricket ball, or due to collision with the advertisement billboards traditionally placed at the boundary [75]. Batsmen and close-in fielders such as the wicketkeeper are also vulnerable to rebound injuries caused as a result of visual inadequacies leading to inappropriate evasive action [76]. In addition, batsmen may also be subjected to muscle tears and strains, whereas wicketkeepers may also experience osteoarthritic changes in the knees due to repetitive squatting [66].

#### Prevention

Major cricket teams hire professional experts such as batting, fielding and bowling coaches to guide the players on injury prevention. In addition, professional cricketers participate in training sessions and warm-up exercises before the match. During the match, batsmen and close-in fielders wear protective clothing such as gloves, helmets, pads and guards in order to prevent impact injuries. Each cricket match has drinks and lunch breaks at regular intervals, which help prevent dehydration and hypoglycemia. Most cricket teams also have a physiotherapist, who provides rehabilitation services in the event of an injury. A psychologist also often accompanies the team.

### Social and economic impact of cricket

Cricket has a vast profile of social and economic effects. The social impact of genres such as “gully cricket” have already been described. High profile cricketers are idolized by fans as role models, especially in the subcontinent [77]. For example, Sachin Tendulkar is highly revered in India, whereas Shahid Afridi enjoys a copious amount of fame in Pakistan. Both of these cricketers are ambassadors for major brands, and enjoy heavy commercial endorsements. The influence enjoyed by certain cricketers has also led them into adopting politics as a career. An example is that of Imran Khan, who currently leads the ‘Pakistan Tehreek-e-Insaf’, which won a substantial number of seats in the 2013 general elections.

In the past century, cricket has seen a transition from an imperial game to a global sport [78]. International Cricket Council (ICC), the body responsible for overseeing cricketing issues, arranges a certain number of cricketing tours as part of its “Future Tours” program. Under this program, teams belonging to different countries are required to play other teams both at home and abroad. This provides ample opportunity for social and cultural diffusion among the players and the spectators, as well as for an exhibition of nationalism. This notion is especially relevant to the Cricket Worldcups, where every 4 years a different country is selected for hosting the event. In addition, county cricket and cricket leagues such as the Indian Premiere League enable players belonging to different countries to play in the same team. This provides a unique opportunity for players, who are otherwise seen as archrivals, to represent the same team thereby unveiling new social dimensions.

Cricket also enjoys a demanding presence in social media circles. Cricket websites such as ESPN Cricinfo® maintain large databases containing match summaries and player profiles, which has tremendously helped in broadening the perspective of the audience. In addition, online forums serve as healthy platforms for cricket-related discussions. Cricket also has a wide following on social networking websites, as shown by the fact that the word “Cricket” has over 75 million likes on Facebook®, and the Indian Cricket Team has over 7 million.

#### Cricket and the capital market

Cricket, like many other sports, has been a medium of commercialization. This is, in part, attributable to globalization of the sport and the consequent increase in both ground and television audience [79]. For instance, television rights for the 2011 Worldcup were bought at $2 billion by ESPN Star Sports® [80]. Cricket matches also exercise a significant impact on the stock markets. In the study conducted by Mishra et al., a loss in a cricket match was found to be associated with a significant downward trend in the stock market in India [81]. The impact of cricketing role models was also demonstrated in this paper, whereby the downward trend was larger when Sachin Tendulkar was playing. The Worldcups also contribute highly to the tourism industry, whereby the profits are not only limited to stadium entrance fees, but also encompass accommodation, meals and shopping [82].

Involvement of large amount of capital in cricket has also led to certain drawbacks. For example, cricket labor migration is a problem that plagues both developed as well as developing countries [83,84]. In addition, gambling poses a continuous threat to the integrity of the gentlemen’s game [85]. Gambling in cricket has been referred to as pathological, as it has led to the birth of crimes such as match fixing and spot fixing, whereby the players receive money for a predetermined outcome of the match [86]. Apart from these, despite the economic benefits of mega-events already mentioned, the economic implications of relocation of cricketing mega-events can be disastrous for developing countries, as seen in the 2011 Worldcup which was relocated from Pakistan leading to the crashing of the stock market [87].

#### Gender influence in cricket

Despite the low physiological requirements of playing cricket compared to other sports, it has been traditionally considered “a man’s game”. However, recent times have seen an increase in the number of female cricketers, with the ICC introducing mega-events for the female gender. Even then, the participation of women is still limited at both administrative and playing levels [88].

### Cricket in Pakistan – interest and limitations

Although the national game of Pakistan is hockey, the majority carries a greater reverence for cricket. In the few lines that follow, we will briefly try to elaborate the implications of previously mentioned health impacts on this particular population.

The government of Pakistan does not impose any limitations on games played on public property. This notion has led to sports being played outside stadiums and courts. On one hand, formats such as “gully cricket” seem to provide the country with an inexhaustible supply of competent sportsmen. However, on the other, such events may lead to irreversible damages being incurred to property, players or passers-by. The political instability prevalent in the country has also plagued sporting activities. Terrorist activities have been rampant and have rendered negative impacts on sports. An attack on the Sri Lankan cricket team saw all major sporting activities being pulled out of Pakistan. These, coupled with allegations of nepotism and corruption, have led to severe crippling of the sports system in the country.

The public health implications of watching sporting events in our setup are also appalling. The association of watching excessive television with metabolic conditions has already been discussed. As per Forbes’ list of world’s “fattest countries”, Pakistan ranks 165 out of 194 countries in stipulations of its overweight population, with a figure of about 22.2% people above age 15, traversing the threshold of obesity [60]. This number is likely to climb, with more and more people adopting the roles of “couch potatoes.” For a nation where tolerance is rarely the order of the day, a loss in a match is followed by unnecessary aggression (especially in gatherings), whereas a win is followed by aerial firing. Such occasions also provide “breeding grounds” for bookmakers, who are often criminal-minded and a social nuisance in any case. It is suggested here that the government should formulate official regulations aimed at maintaining a balance between entertainment and health, before this problem gets out of control and difficult to manage.

## Conclusion

In view of the above discussion, cricketing activities can be considered as being advantageous to both physical and mental health. However, in order to derive maximum benefits from sporting activities, an optimal combination of type, frequency and intensity is almost invariably required; a clear consensus exists that regular physical activity of at least 30 minutes of moderate intensity can guarantee a healthy lifestyle. Similarly, although severe health concerns exist for major cricketing events, their cultural significance cannot be undermined. Health and harmony prevail as a result of such events, and their organization brings economic stability to a country. However, efforts should be made to regulate commercials and advertisements that promote inappropriate or unhealthy content. Similarly, proper security protocols should also be strictly followed in order to ensure a substantial audience. Keeping the above in mind, we can successfully conclude that the glimmer of the pros of cricket may be suppressed by its cons, most of which are indirect and can be controlled with proper implementation of international guidelines.

## Competing interests

The authors declare that no competing interests exist. The authors did not receive any financial support or grant for this study.

## Authors’ contributions

AS conceived the topic and was involved in drafting the initial manuscript. SAA, AS, SA and SSA were involved in critically revising the manuscript, listed in decreasing order of their contributions. The authors have read and approved the manuscript.
